# Supplementary material for: Neuronal Dynamics of Pain in Parkinson’s Disease
Source: Brain Sci. 2021 Sep 16;11(9):1224. doi: 10.3390/brainsci11091224 (PMC8468705; doi:10.3390/brainsci11091224)
Supplement: Supplementary file 1 [file brainsci-11-01224-s001.zip › brainsci-1316574-supplementary.pdf]

Figure S1

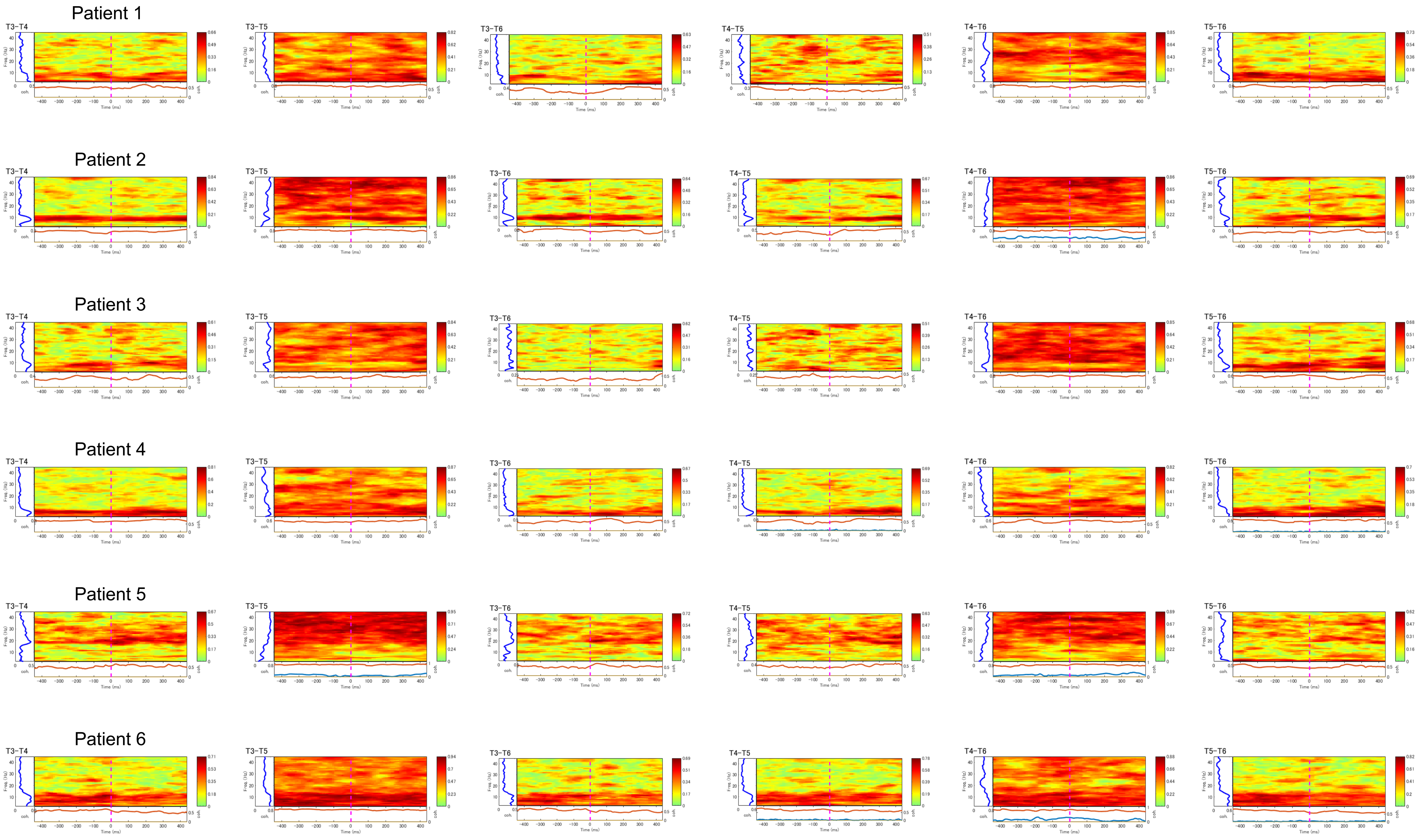

(continued)

Figure S1
(continued)

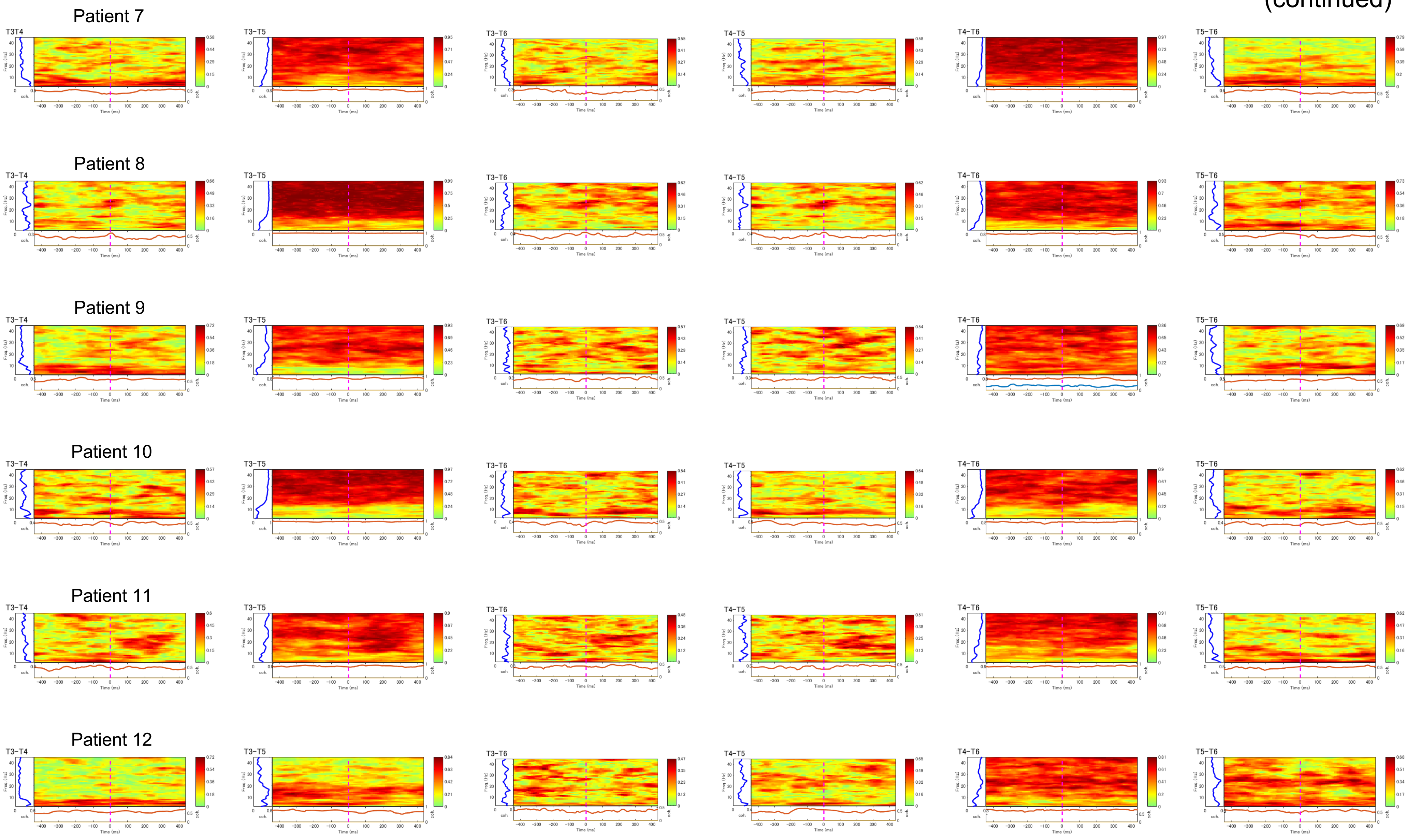

(continued)

Figure S1
(continued)

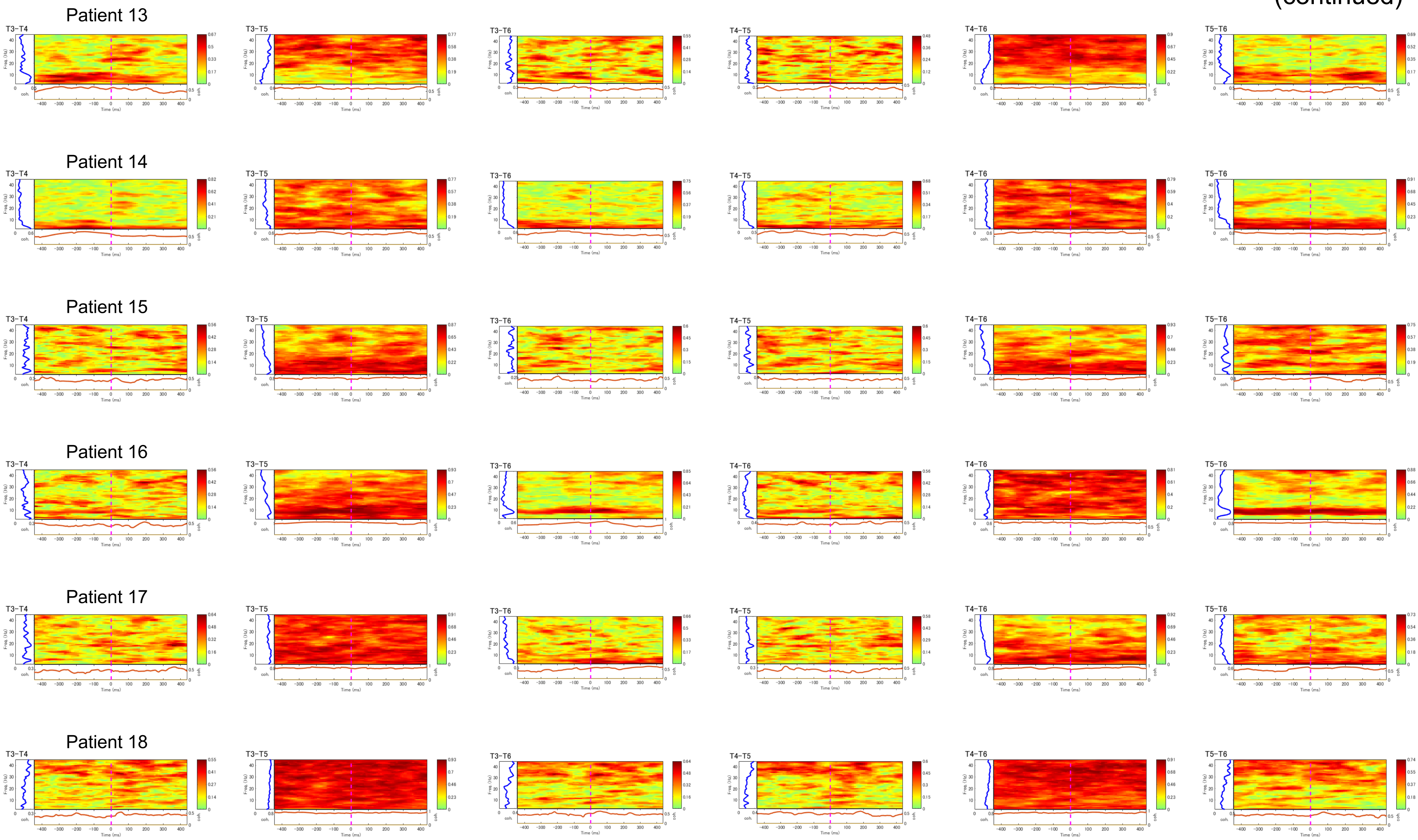

(continued)

Figure S1
(continued)

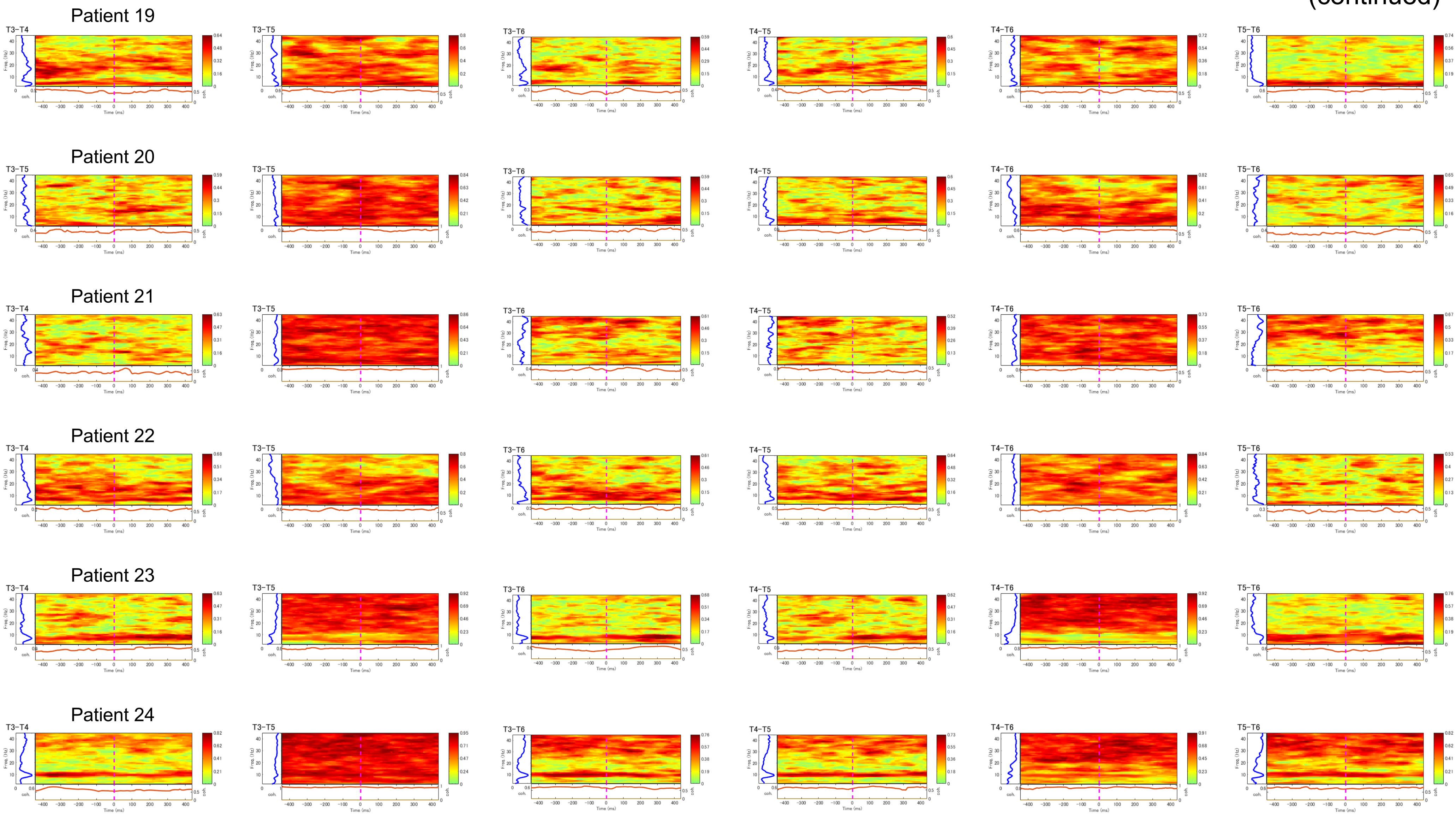

Figure S1: Time frequency and coherence analysis on EEG data. Each plot shows the coherence value across temporal regions (T3-T4, T3-T5, T3-T6, T4-T5, T4-T6, T5-T6) at each time (horizontal line) and each frequency (vertical line) of all 24 patients.
